# To Save from Drowning

**Published:** 1879-08

**Authors:** 


### TO SAVE FROM DROWNING.

Never approach a drowning person from the front, but take him from behind by the hair, and never allow him to grasp any part of your body if you can possibly avoid it. Should he do so sink at once to the bottom, when he will release his hold on you. Only a good swimmer should attempt to rescue a drowning man in deep water; for a novice to try it is to imperil a second life with little chance of saving the first. Better hasten to secure a rope or pole, or plank, which throw to the person in danger. This will give him a better chance of escape. If possible, let one going to the rescue have a rope tied under his own arms and held by some one on shore, or tied securely to some firm object. Young swimmers should use great caution.

